# Microbiota does not influence tumor development in two models of heritable cancer

**DOI:** 10.1128/mbio.03866-24

**Published:** 2025-02-19

**Authors:** Jessica Spring, Sandeep Gurbuxani, Tatyana Golovkina

**Affiliations:** 1 Committee on Microbiology, University of Chicago, Chicago, Illinois, USA; 2 Department of Pathology, University of Chicago, Chicago, Illinois, USA; 3 Department of Microbiology, University of Chicago, Chicago, Illinois, USA; 4 Committee on Immunology, University of Chicago, Chicago, Illinois, USA; 5 Committee on Genetics, Genomics and System Biology, University of Chicago, Chicago, Illinois, USA; Dana-Farber Cancer Institute, Boston, Massachusetts, USA

**Keywords:** microbiota, cancer, gut commensal bacteria, heritable cancer, genetic predisposition to cancer

## Abstract

Microbial impact on tumorigenesis of heritable cancers proximal to the gut is well-documented. Whether the microbiota influences cancers arising from inborn mutations at sites distal to the gut is undetermined. Using two models of heritable cancer, *Trp53*-deficient mice and Wnt1-transgenic mice, and a gnotobiotic approach, we found the microbiota to be inconsequential for tumor development. This work furthers our understanding of the degree of the microbial impact on tumor development.

**IMPORTANCE:**

The influence of the microbiome on the development of cancer is well-documented with many if not all published studies reporting either a positive or a negative impact. None of the published studies, however, presented data on the influence of the microbiome on the development of heritable cancer. We find that the microbiota has no influence on cancer development in two models of spontaneous cancers driven by germline *Trp53* deficiency and constitutive Wnt1 signaling.

## INTRODUCTION

Cancer arises from sporadic or inborn mutations within oncogenes, tumor suppressor genes, or the regulatory regions that control their expression (1). Sporadic mutations occur randomly during normal cell division or emanate from radiation, chemical carcinogens, or viral infections (2, 3). On the other hand, inborn mutations are inherited and can cause familial cancers (4, 5). The influence of the microbiota on carcinogenesis stemming from spontaneous mutations has been well-documented by us and other researchers (6–8). The influence of commensal bacteria on the progression of transplantable tumors has also been well-documented (9, 10). Furthermore, the development and progression of hereditary colorectal cancer, often caused by mutations in tumor suppressor genes such as adenomatous polyposis coli (APC), have also been found to be influenced by the microbiota (6, 11). However, the potential role the microbiota plays in carcinogenesis of heritable cancers emerging distal to the gut and other non-sterile organs remains understudied.

To determine the influence of the microbiota on hereditary cancer, it is essential to re-derive mice with cancer-predisposed mutations as germ-free (GF) and monitor the incidence and latency of tumor development in these GF mice and their specific pathogen-free (SPF) counterparts.

Using this approach, we demonstrate that the microbiota does not impact tumor development in *Trp53*-deficient and Wnt1-transgenic mice, two well-known mouse models of heritable cancer.

## MATERIALS AND METHODS

### Mice

The following mice used in this study were bred and maintained at the animal facility of the University of Chicago. B6.129S2-*Trp53^tm1Tyj^/J* (B6.Trp53^−/−^) and FVB.Cg-Tg(Wnt1)1Hev/J (FBV.wnt1Tg) were purchased from the Jackson Laboratory. Specific pathogen-free (SPF) and germ-free (GF) mice were housed in the animal resource center under the supervision of the University of Chicago veterinarians. Males and females used in the studies were littermates produced by the same mothers (*Trp53^−/−^* or Wnt1 transgenic, respectively) and, thus, had similar microbiome derived from their mothers. Both *Trp53^−/−^* males and females (at approximately 50:50 ratio) and virgin Wnt1 hemizygous transgenic females were used for tumor monitoring. Males and females were housed based on genders, with 2–5 mice per cage.

The animal facility at the University of Chicago is an AAALAC-accredited facility. Animals were provided food and water *ad libitum*. SPF mice were fed on irradiated standard 6% fat NIH-31 mouse chow. Germ-free mice were fed on 6% fat 5K67 mouse chow, which is similar in ingredients and nutrient composition to NIH-31 chow but is fortified with vitamins to compensate for loss during autoclaving required for sterilization. Each cage has nestlets as environmental enrichment. Both SPF and GF mice were checked for tumors daily, and animals bearing detectable tumors (<5 mm) were euthanized. Mice were also checked for hunched posture and pale look (these are two phenotypes that segregate with leukemia development). Mice looking pale and having hunched posture were euthanized. Mice were euthanized by forced CO_2_ inhalation delivered in a sealed chamber from a cylinder with compressed CO_2_ gas. Cervical dislocation was performed as a second method for euthanasia. These methods are consistent with the recommendations of the Panel on Euthanasia of the American Veterinary Medical Association.

### Monitoring sterility of germ-free and mouse pathogens in specific pathogen-free mice

B6.Trp53^−/−^ and FBV.wnt1Tg mice were re-derived as GF at Taconic Biosciences and housed in isolators in the gnotobiotic facility at the University of Chicago. Assessment of GF isolator sterility was conducted as previously described (12). Briefly, fecal pellets collected weekly from isolators were subjected to DNA extraction using a bead beating/phenol–chloroform extraction protocol. A single fecal pellet was placed in an autoclaved 2 mL screw-cap tube containing 0.1 mm zirconium beads, along with 500 µL of 2× buffer (filter sterilized 200 mM NaCl, 200 mM Tris, 20 mM EDTA), 210 µL of 20% SDS, and 500 µL phenol:chloroform. The tube was bead beat for 2 minutes and then centrifuged at 8,000 rpm at 4°C for 3 minutes. The aqueous phase was placed in a new Eppendorf tube, and 500 µL of phenol:chloroform was added. The tube was centrifuged at 13,000 rpm at 4°C for 3 minutes. The aqueous 400 µL phase was supplied with 40 µL of 3 M sodium acetate (pH 7) and 400 µL of −20°C isopropanol and then spun at 13,000 rpm at 4°C for 10 minutes. The supernatant was discarded, and 500 µL of 80% ethanol at −20°C was added. The sample was spun at 13,000 rpm at 4°C for 5 minutes. The supernatant was again dumped, and the sample was vacuum dried for 10 minutes. The pellet was resuspended in 1 mL of sterile water and left overnight at 4°C. Primers that broadly hybridize to bacterial 16S rRNA gene sequences (5′GACGGGCGGTGWGTRCA3′ and 5′AGAGTTTGATCCTGGCTCAG3′) were used to amplify isolated DNA. In addition, brain heart infusion, nutrient, and Sabouraud broth containing tubes were inoculated with fecal pellets collected from the same GF cages, from SPF cages (positive control) and with sterile saline (negative control), and incubated at either 37°C or 42°C in both aerobic and anaerobic conditions to test for bacterial and fungal contamination. Cultures were monitored for 5 days until deemed negative. All mice were tested once a week and at closing. All animals remained sterile throughout the duration of the studies and at closing.

SPF mice were routinely tested for mouse-excluded pathogens, such as mouse hepatitis virus, Sendai virus, pneumonia virus of mice, Theiler’s mouse encephalomyelitis virus, reovirus-3, mouse rotavirus, *Ectromelia virus*, lymphocytic choriomeningitis virus, polyoma virus, mouse cytomegalovirus, mouse adenovirus, K virus, mouse thymic virus (MTV), hantavirus, lactate dehydrogenase elevating virus (LDEV), parvoviruses, minute virus of mice, mouse parvovirus, *Mycoplasma pulmonis*, *Salmonella* spp., *Citrobacter rodentium*, *Clostridium piliforme*, *Streptobacillus moniliformis*, *Filobacterium rodentium*, *Corynebacterium kutscheri*, pinworms (*Syphacia* spp., *Aspiculuris tetraptera*), fur mites (*Myobia musculi*, *Myocoptes musculinus*, *Radfordia affinis*, *Psoregates simplex*), and *Giardia* spp.

### Histology

Visible tumors were excised from SPF and GF *Trp53*-deficient and Wnt1-transgenic mice and fixed in Telly’s fixative. Tumor type was determined based on morphologic assessment of hematoxylin and eosin stained 4 µm sections. Classification of the malignancies was based on well-recognized morphological patterns. A diagnosis of lymphoma was rendered when the tumor presented as a discrete mass with discohesive intermediate-sized tumor cells. Sarcomas were more cohesive, and cytomorphology was notable for presence of spindle-shaped cells mixed with larger pleomorphic cells with variable nuclear lobation. Carcinomas showed lumen formation, a characteristic of adenocarcinomas.

## RESULTS

**Fig 1:**
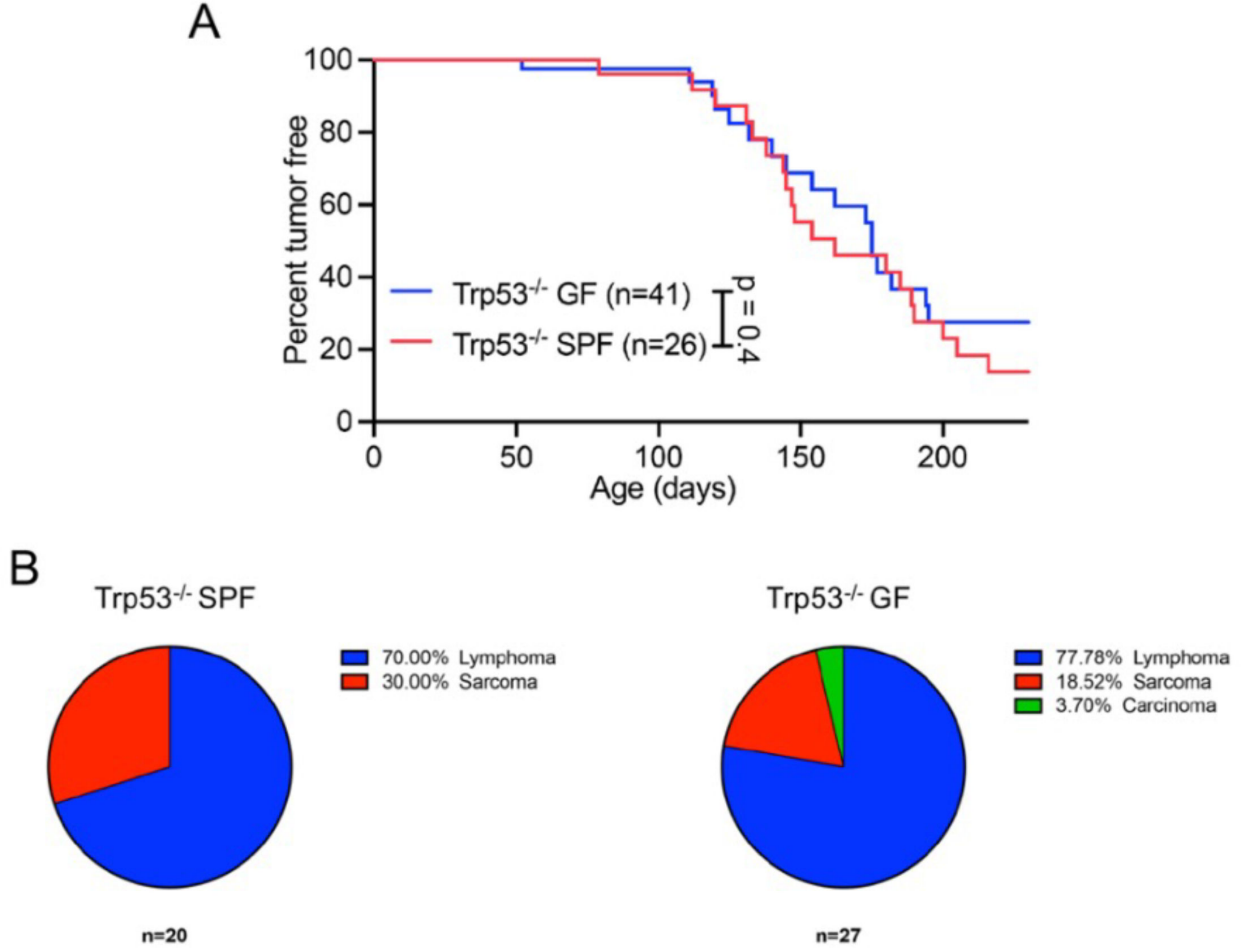
Microbiota does not impact the latency and incidence of tumor development in *Trp53^−/−^* mice. (**A**) *Trp53^−/−^* SPF and GF mice were monitored for tumor development. (**B**) Proportion of SPF *Trp53^−/−^* mice that develop various forms of tumors in SPF (left) and GF (right) mice. *n*, number of mice used. Of 26 SPF *Trp53^−/−^* mice, 15 were males and 11 were females, whereas of 41 GF *Trp53^−/−^* mice, 30 were males and 21 were females. Twenty of 26 SPF tumors were histologically analyzed. Of those, 14 were identified as lymphomas and six as sarcomas. Of 41 GF tumors, 27 were histologically analyzed. Of those, 21 were identified as lymphomas, five as sarcomas, and one as carcinoma. *P* values calculated using Mantel-Cox test (**A**).

The first model tested was tumor suppressor p53 (encoded by *TRP53* in humans and *Trp53* in mice) deficiency. *TRP53* is crucial for enabling response to cell stress, such as DNA damage, by arresting cell cycle or inducing apoptosis (13). Mutations within *TRP53* are the most frequently observed mutations in human cancers (14). In addition, germline mutations in *TRP53* are associated with Li-Fraumeni syndrome, a cancer with familial predisposition (15). The vast majority of tumors developing in *Trp53^−/−^* mice are lymphomas/leukemia and soft tissue sarcomas, with approximately 50% of mice developing cancer by 3–4 months (16). Therefore, the survival rate and tumor development were compared in male and female *Trp53^−/−^* mice housed under SPF and GF conditions for 1 year. Moribund mice or mice displaying overt tumors were euthanized and examined by necropsy; their tumors were analyzed by histopathology. The frequency of tumor development in GF *Trp53*-deficient mice was not significantly different to that observed in SPF *Trp53*-deficient mice (Fig. 1A). Furthermore, all SPF mice and the majority of GF *Trp53*-deficient mice developed lymphomas or sarcomas (Fig. 1B) in agreement with the previously published data (17). One GF *Trp53*-deficient mouse developed carcinoma, a cancer type less frequently associated with this mouse model (16).

**Fig 2:**
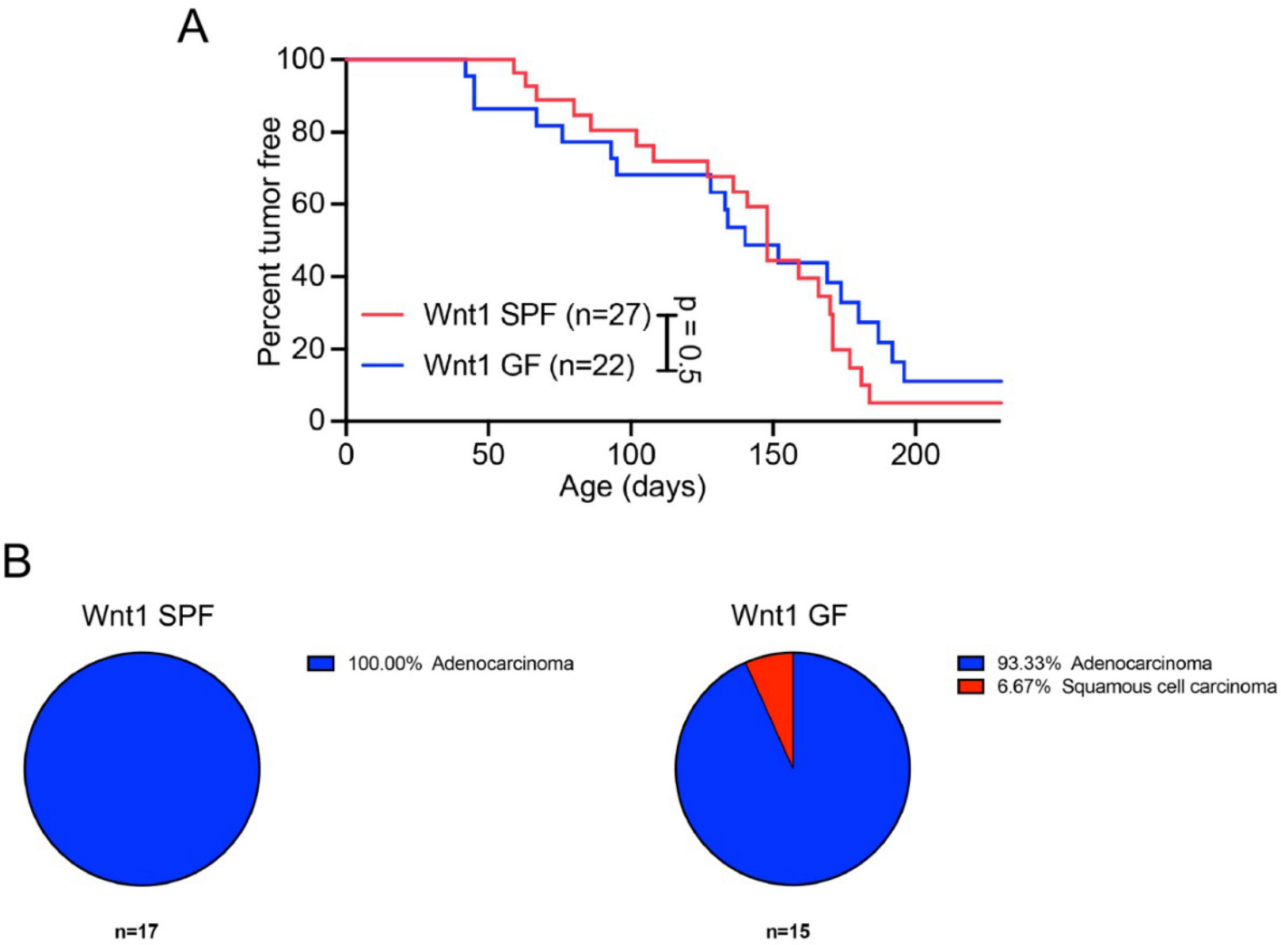
The microbiota does not influence the latency and incidence of tumor development in Wnt1-transgenic mice. (**A**) Virgin Wnt1-transgenic SPF and GF females were monitored for tumor development. (**B**) Proportion of Wnt1-transgenic mice that develop various types of tumors in SPF (left) and GF (right) mice. *n*, number of mice used. Seventeen of 27 SPF tumors were histologically analyzed and identified as adenocarcinomas. Fifteen of 22 GF tumors were histologically analyzed, 14 were identified as adenocarcinomas, and one as carcinoma. *P* values calculated using Mantel-Cox test (**A**).

To further understand the influence of the microbiota on tumor development stemming from genetic predisposition, we also tested Wnt1-transgenic mice, a model for mammary carcinoma development. Wnt1 canonically controls cell proliferation by increasing and stabilizing cytosolic β-catenin, which then translocates to the nucleus and facilitates the expression of genes, including cell cycle regulators c-myc and cyclin D1 (18). Atypical Wnt1 expression under the mammary tumor virus promoter (MMTV-Wnt1) within Wnt1-transgenic mice induces mammary adenocarcinomas (18, 19). Mammary glands from virgin Wnt1 hemizygous females resemble hormonally stimulated glands from pregnant mice. Adenocarcinomas developed in virgin females between 3 and 7 months, and more rarely in males. Tumors arose stochastically, indicating additional events are required for neoplasia development (18, 19). GF MMTV-Wnt1 transgenic virgin females developed tumors with similar latency and incidence compared to SPF MMTV-Wnt1 transgenic virgin females (Fig. 2A). Wnt1-transgenic females in both housing conditions primarily developed adenocarcinomas, the archetypal tumor type characteristic of this model (Fig. 2B). One mouse developed squamous cell carcinomas (Fig. 2B), which had never been seen before in SPF Wnt1-transgenic mice (19).

## DISCUSSION

Prior studies, including our study investigating tumors resulting from spontaneous mutations, have defined a significant role for the microbiota in the development of colorectal cancer (6, 8) and virally induced leukemia (7). In contrast to these studies, the latency and incidence of hereditary lymphoma/sarcoma in *Trp53^−/−^* mice and mammary gland adenocarcinomas in Wnt1-transgenic mice were unaffected by the microbiota (Fig. 1A and 2A). These data suggest that unlike spontaneous malignancies, the development of cancer arising from specific genetic predispositions in *Trp53^−/−^* and Wnt1-transgenic mice is not influenced by the microbiota.

**Fig 3:**
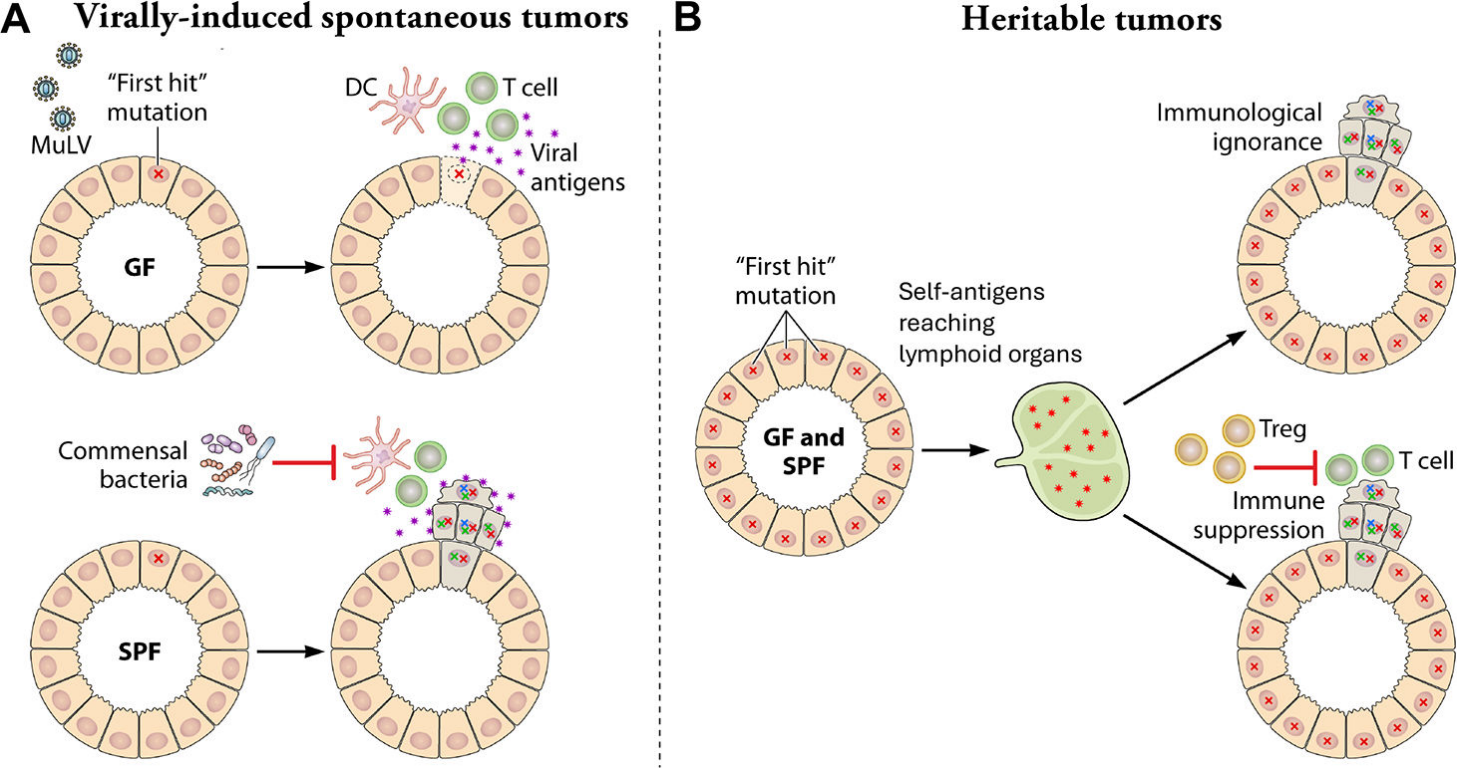
Intrinsic dissimilarities between spontaneous (virally induced) and heritable tumors which could explain differential dependence on the microbiota. (**A**) Virally induced tumors, such as MuLV-induced leukemia are initiated from rare cells with proviral integration next to the cellular proto-oncogene (“first hit” mutation, shown as red X). In MuLV-infected germ-free (GF) mice, viral antigens expressed by precancerous cells stimulate a strong immune response suppressing leukemia development (acquisition of additional mutations required for tumor progression). In MuLV-infected specific pathogen-free (SPF) mice, commensal bacteria suppress the immune response against viral antigens enabling acquisition of additional mutations (shown as green and blue XX) and progression to leukemia. Data shown in the figure are from reference 7. (**B**) The “first hit” mutation in heritable cancer is present since birth. Self-antigens expressed in excess by tumor cells may cause either immunological ignorance or immune suppression leading to tumor development regardless of the presence of the microbiota. DC, dendritic cell; Treg, T regulatory cell.

The discordance in these observations may be explained by the inherent differences between tumors that develop spontaneously and the tumors that develop as a result of genetic predispositions, such as in *Trp53^−/−^* and Wnt1-transgenic mice. Spontaneous tumors, induced by viruses or carcinogens, or due to random mutations, derive from a limited number of cells in which oncogenes are upregulated, tumor suppressor genes are nullified, or their regulatory regions are mutated. These rare tumor cells must escape the immune response targeting tumor-specific antigens, such as viral antigens or neoantigens. Previously, we showed that some commensal bacteria promote the development of murine leukemia virus (MuLV)-induced leukemia, a type of spontaneous tumors induced by the insertional activation of cellular proto-oncogenes (20), by suppressing the adaptive immune response through induction of several negative immune regulators (7). The negative immune regulators, such as Serpinb9b and Rnf128, were upregulated in MuLV-infected leukemia-susceptible SPF mice, but not in MuLV-infected leukemia-resistant GF mice (7) (Fig. 3A). Serpinb9b is a serine protease inhibitor that acts on and suppresses granzyme M produced by cytotoxic cells (21). Rnf128 is a ubiquitin ligase that has been shown to ubiquitinate CD3 and CD40L expressed on T cells, leading to these molecules degradation and, thus, T cell unresponsiveness (22, 23). Therefore, tumor-specific T cell-mediated responses were highly effective in GF mice but were suppressed by the microbiota in SPF mice, thus promoting virally induced leukemia development (7). In other models of sporadic tumors, such as hepatocellular and colorectal cancer, gut bacteria and their byproducts affected the progression of cancer through a variety of means including damaging DNA and promoting inflammation (6, 24–26).

In contrast to spontaneous tumors, inborn mutations predisposing to cancer are ubiquitous. Self-antigens expressed in excess by tumor cells may cause either immunological ignorance or immune suppression leading to tumor development (27–29). These processes alone drive tumorigenesis regardless of the presence or absence of the microbiota (Fig. 3B).

Although the peripheral immune organs, such as Peyer’s patches, are underdeveloped in GF mice compared to SPF mice (30), their immune system is not compromised and responds to immune challenges, such as immunization similar to SPF mice (12, 31). As mentioned above, GF mice are also capable of mounting T cell-mediated responses preventing development of virally induced tumors (7). Since there was no difference in the latency and incidence of tumors developed between GF and SPF mice (Fig. 1A and 2A), we saw no reason to evoke the question of incompetence of the immune system of GF mice as a factor contributing to tumor development.

Our results deviate from the results reported by Yamamoto et al. (32), which suggested that the microbiota can influence lymphoma development in a model of heritable cancer. Yamamoto et al. utilized mice deficient in ataxia telangiectasia mutated (ATM) gene that are genetically predisposed to lymphoid cancers. In humans, mutations in this gene cause ataxia telangiectasia, an autosomal recessive disease that is also associated with a higher incidence of lymphoid cancers (33). ATM is activated by double-stranded DNA breaks and functions upstream of *Trp53* by phosphorylating *Trp53*, thus inducing either cell cycle arrest or apoptosis (34). Yamamoto et al. found that *ATM*-deficient mice exhibited increased lymphoma latency when housed in SPF conditions and supplied with sterile food, water, and bedding in comparison to *ATM*-deficient mice housed in SPF conditions with non-autoclaved supplies (32). They also found that *ATM*-deficient mice gavaged with a restricted microbiota following antibiotic treatment had an increased lifespan compared to mice gavaged with a conventional microbiota after antibiotic treatment (32). The authors of the studies suggested that the microbiota, specifically bacteria, affected lymphomagenesis in *ATM^−/−^* mice. The enrichment of *Lactobacillus johnsonii* (*L. johnsonii*) (32) and some metabolites in mice with the restricted microbiota (35) were proposed to have an anti-tumor effect. However, the direct impact of *L. johnsonii* and the metabolites on lymphoma development was never scored. It is possible that the shift in unidentified factor(s) different from the commensal bacteria altered lymphomagenesis in *ATM^−/−^* mice in these experimental settings. The current standards in the microbiota field require the usage of GF (36), which we used in our studies. Although we have not used *ATM^−/−^* mice, we exploited better breeders, specifically *Trp53^−/−^* mice. As ATM functions upstream of Trp53 (34), *Trp53^−/−^* mice can be viewed as a proxy for *ATM^−/−^* mice.

It is well appreciated that the microbiota is dependent on factors such as husbandry (37), genetic background (38), and diet (39), and thus, is highly likely to be different between different facilities and among mice from different genetic backgrounds. Over the past two decades, many research groups have collected survival data using SPF *Trp53^−/−^* (40–42) and Wnt1-transgenic mice (19, 43) at different facilities and often using mice of different genetic backgrounds. In all published cases, the incidence and latency of the tumor development in mice from these two models were identical to the incidence and latency we observed in our studies. Specifically, the incidence of observable tumors was reported in 41/49 (40), 19/23 (41), and 125/125 (42) of *Trp53^−/−^* B6J mice during 9 months of monitoring, which is highly congruent with our findings (Fig. 1A). These mice predominantly developed lymphomas, followed by sarcomas, parallel to our study (Fig. 1B). Investigations into the latency and incidence of mammary adenocarcinomas in Wnt1-transgenic mice determined that roughly 80% of mice had developed tumors by 7 months of age (19, 43), similar to our findings (Fig. 2A). These studies indicate that variations in microbiomes in different SPF colonies are unlikely to influence the tumor development in *Trp53^−/−^* and Wnt1-transgenic mice.

In conclusion, utilizing *Trp53^−/−^* and Wnt1-transgenic mice as well as accepted standards for microbiome studies, we found no contribution of microbiota to tumor development in these two models.

### Limitation

This study demonstrates the microbiota does not impact tumor development in two models of heritable cancer, such as *Trp53^−/−^* and Wnt1-transgenic mice. However, these results may not be generalizable to tumors arising from other genetic predispositions.
